# Targeting Acid Ceramidase to Improve the Radiosensitivity of Rectal Cancer

**DOI:** 10.3390/cells9122693

**Published:** 2020-12-15

**Authors:** Rachael E. Clifford, Naren Govindarajah, David Bowden, Paul Sutton, Mark Glenn, Mahnaz Darvish-Damavandi, Simon Buczacki, Ultan McDermott, Zdzislaw Szulc, Besim Ogretmen, Jason L. Parsons, Dale Vimalachandran

**Affiliations:** 1 Cancer Research Centre, Department of Molecular and Clinical Cancer Medicine, University of Liverpool, 200 London Road, Liverpool L3 9TA, UK; ng104@liv.ac.uk (N.G.); dbowden@nhs.net (D.B.); Paulsutton01@doctors.org.uk (P.S.); m.a.glenn@liv.ac.uk (M.G.); j.parsons@liv.ac.uk (J.L.P.); 2 Nuffield Department of Surgical Science, University of Oxford, Oxford OX3 7DQ, UK; mahnaz.darvishdamavandi@nds.ox.ac.uk (M.D.-D.); simon.buczacki@nds.ox.ac.uk (S.B.); 3 Wellcome-MRC Cambridge Stem Cell Institute, University of Cambridge, Cambridge CB2 0AW, UK; 4 Oncology R&D, AstraZeneca, Cambridge CB2 0AW, UK; um1@sanger.ac.uk; 5 Department of Biochemistry and Molecular Biology, Medical University of South Carolina, Charleston, SC 29425, USA; szulcz@musc.edu (Z.S.); ogretmen@musc.edu (B.O.); 6 Clatterbridge Cancer Centre NHS Foundation Trust, Clatterbridge Road, Bebington CH63 4JY, UK; 7 The Countess of Chester Hospital, Liverpool Road, Chester CH2 1UL, UK

**Keywords:** acid ceramidase, ionizing radiation, LCL521, rectal cancer

## Abstract

Previous work utilizing proteomic and immunohistochemical analyses has identified that high levels of acid ceramidase (AC) expression confers a poorer response to neoadjuvant treatment in locally advanced rectal cancer. We aimed to assess the radiosensitising effect of biological and pharmacological manipulation of AC and elucidate the underlying mechanism. AC manipulation in three colorectal cancer cell lines (HT29, HCT116 and LIM1215) was achieved using siRNA and plasmid overexpression. Carmofur and a novel small molecular inhibitor (LCL521) were used as pharmacological AC inhibitors. Using clonogenic assays, we demonstrate that an siRNA knockdown of AC enhanced X-ray radiosensitivity across all colorectal cancer cell lines compared to a non-targeting control siRNA, and conversely, AC protein overexpression increased radioresistance. Using CRISPR gene editing, we also generated AC knockout HCT116 cells that were significantly more radiosensitive compared to AC-expressing cells. Similarly, two patient-derived organoid models containing relatively low AC expression were found to be comparatively more radiosensitive than three other models containing higher levels of AC. Additionally, AC inhibition using carmofur and LCL521 in three colorectal cancer cell lines increased cellular radiosensitivity. Decreased AC protein led to significant poly-ADP ribose polymerase-1 (PARP-1) cleavage and apoptosis post-irradiation, which was shown to be executed through a p53-dependent process. Our study demonstrates that expression of AC within colorectal cancer cell lines modulates the cellular response to radiation, and particularly that AC inhibition leads to significantly enhanced radiosensitivity through an elevation in apoptosis. This work further solidifies AC as a target for improving radiotherapy treatment of locally advanced rectal cancer.

## 1. Introduction

Neoadjuvant chemoradiotherapy (CRT) remains the mainstay of treatment for locally advanced rectal cancer [1]. However, response to such treatment remains both variable and unpredictable and has led to an ongoing drive to develop novel prognostic and/or therapeutic biomarkers/molecular targets [2]. Successful response to CRT not only improves the likelihood of surgical resection of the tumour with clear margins, but if the pathological response is significant there is an associated improvement in overall long-term survival. Unfortunately, only 12% of patients with locally advanced rectal cancer experience a complete pathological response [3], and a significant proportion develop disease progression locally and/or systemically during CRT.

The primary mechanism by which CRT delivers its effect on cancer cells is through the induction of DNA damage, particularly via DNA double strand breaks [4]. However, cancer cells are equipped with cellular DNA damage response mechanisms that typically repair the DNA damage and which can promote radioresistance. Such resistance is also thought to be mediated by cellular sub-populations, particularly cancer stem cells, that possess properties such as efficient DNA damage repair and self-renewal [5]. The combination of radiotherapy with an agent that enhances tumour killing is well-recognised, and typically in rectal cancer a cytotoxic radiation-chemical reaction is employed, most often using a fluorine analogue such as 5-fluorouracil (5-FU) [6]. The exact molecular mechanisms by which 5-FU exerts its radiosensitising effects are not fully understood, but are in part thought to be through its production of fluorodeoxyuridine (FdUrd) and its subsequent effects on both RNA function and DNA repair post irradiation [7]. Despite much effort, there are at present no other novel or more effective radiosensitising agents available for rectal cancer that can improve on currently observed response rates [6].

We have previously performed temporal proteomic analysis of patients undergoing CRT through serial rectal biopsies with associated oncological outcome data [8]. Using this approach, we uniquely identified acid ceramidase (AC) as being over-expressed in patients who responded poorly to CRT. AC is a lysosomal cysteine hydrolase encoded by the *asah1* gene, which catalyzes the conversion of ceramide into fatty acid and sphingosine [9]. Ceramide plays a central role in response to cellular stress, and its synthesis and accumulation can mediate cell death through a number of mechanisms, including apoptosis and autophagy. In addition to lowering ceramide levels, this reaction also produces sphingosine-1-phosphate (S1P), which can alternatively lead to an increase in cellular proliferation. This tightly regulated balance between ceramide and S1P is increasingly thought to play a key role in cancer development and progression, and AC has been identified as a potential novel therapeutic target which is overexpressed in a number of human cancers, such as prostate and head and neck cancers [10]. Inhibition of AC has been shown to both inhibit cancer cell formation in melanoma cell lines, but importantly has also been demonstrated to sensitize glioblastoma and prostate cancer cell lines to radiation [11,12,13,14]. Historically, pharmacological inhibition of AC has been achieved through the off-target effects of traditional cytotoxic agents [15,16], although the side-effect profile of many of these drugs has limited their use. Specific ceramide analogs, such as B13, will effectively inhibit AC in vivo but have limited in vitro use due to their inability to access the lysosomal compartment where AC resides [17]. Development of structurally altered B13 prodrugs, such as LCL521, are however, able to provide novel, specific subcellular targeted delivery of B13 and thus achieve AC inhibition [18].

In this study, we now further analyse the impact of AC in mediating radioresistance of colorectal cancer cell lines in vitro through both biological (siRNA and CRISPR) manipulation and pharmacological inhibition of AC. To this effect, we demonstrate that targeting AC can enhance the radiosensitivity of colorectal cancer cells through promoting cellular apoptosis.

## 2. Materials and Methods

### 2.1. Cell Culture

Colorectal cancer cell lines (HCT116, HT29, LIM1215, MDST8, GEO, NCI-H716; Wellcome Sanger Institute, Cambridge, UK) were all short tandem repeat (STR) profiled and tested free of mycoplasma. p53^−/−^ HCT116 cell line was kindly provided by Prof B. Vogelstein. HCT116 cells were cultured in Dulbecco’s modified Eagle’s medium (DMEM; Sigma-Aldrich, Gillingham, UK) supplemented with 10% fetal bovine serum, 1% L-glutamine, 1% Penicillin-streptomycin and 1% non-essential amino acids at 37 °C in 5% CO_2_. All other cells were cultured in Roswell Park Memorial Institute (RPMI; Sigma-Aldrich, Gillingham, UK) with the same supplements, but additionally with 10 mM hydroxyethyl piperazineethanesulfonic acid (Hepes). siRNA knockdown of *asah1* (using a single siRNA sequence; D-005228-03, Horizon Discovery Ltd., Cambridge, UK) or using a non-targeting control siRNA (AllStars Negative Control siRNA; Qiagen, Manchester, UK) was performed using Lipofectamine RNAiMAX (Life Technologies, Paisley, UK) for 48 h. The mammalian overexpression plasmid for *asah1* was kindly provided by Dr D. Krainc, and transfection with this plasmid was performed for 24 h using Lipofectamine 2000 (Life Technologies, Paisley, UK). Carmofur and 5-FU were purchased from Sigma-Aldrich (Gillingham, UK). Organoids were characterised and kindly gifted by The Sanger Institute (Cambridge, UK). They were embedded in 40 μL of Matrigel (Corning, NY, USA) in 24-well plates and grown in Intesticult™ Organoid Media (StemCell, Cambridge, UK) supplemented with 1% Penicillin-streptomycin and 10 μM Y27632 dihydrochloride, Rho kinase (ROCK) inhibitor. For passaging organoids, matrigel domes were submerged in 1 mL TrypLE 1X (Gibco, ThermoFisher Scientific, Waltham, MA, USA) and incubated at 37 °C for 10 min. Aspirated cells were suspended in cold PBS and centrifuged at 1200 rpm for 5 min. The pellet was then resuspended in Matrigel and plated onto a pre-warmed 24-well plate.

### 2.2. Preparation of Whole Cell Extracts and Immunoblotting

Whole cell extracts (WCE) were prepared and immunoblotting analysis performed using the Odyssey Image Analysis System (Li-Cor Biosciences, Cambridge, UK), as previously described [19,20]. Primary antibodies raised against ASAH1 (1:500) (BD Biosciences, Wokingham, UK), poly-ADP ribose polymerase-1 (PARP-1) (1:5000) (Santa Cruz Biotechnology, Heidelberg, Germany) or actin/tubulin (1:20,000) (Sigma-Aldrich, Gillingham, UK), along with Alexa Fluor 680 or IR Dye 800 secondary antibodies (1:20,000) (Li-Cor Biosciences, Cambridge, UK) were used for detection.

### 2.3. asah1 mRNA Analysis

Affymetrix Human Genome U219 microarray data were analysed on the Human Genome U219 96-Array Plate using the Gene Titan MC instrument (Affymetrix, ThermoFisher Scientific, Waltham, MA, USA). The robust multi-array analysis (RMA) algorithm was used to establish intensity values for each of 18,562 loci. Raw data were finally deposited in ArrayExpress (accession number: E-MTAB-3610). The RMA processed dataset is available at http://www.cancerrxgene.org/gdsc1000/ [21].

### 2.4. Detecting AC Using ELISA

A sandwich enzyme-linked immunosorbent assay (ELISA) kit (Cloud-Clone Corporation, Houston, TX, USA) was used to establish a dose response relationship for carmofur and LCL521 in inhibiting AC antibody binding in colorectal cancer cell lysates. AC standards and cell lysates were added to a 96-well plate pre-coated with a biotin-conjugated AC specific antibody and incubated with avidin conjugated to Horseradish Peroxidase. After addition of the tetramethylbenzidine substrate, the colour change was assessed spectrophotometrically at 450 nm and AC detection levels in the cell lysates determined relative to the levels in the standards.

### 2.5. Clonogenic Assays

Clonogenic assays were performed as previously described [22,23], following irradiation with a CellRad X-ray irradiator (Faxitron Bioptics, Tucson, AZ, USA). For drug treatments, cells were treated as a monolayer for 2 h prior to irradiation and using DMSO as a vehicle only control. Cells were trypsinized, counted and plated into 6-well plates and colonies allowed to grow for 10–14 days. Specifically for LCL521 treatments, cells were treated with the drug for a further 24 h post-irradiation. Colonies were counted using a GelCount colony counter (Oxford Optronix, Oxford, UK), and relative colony formation (surviving fraction) was expressed as colonies per treatment level versus colonies that appeared in the untreated control. All experiments were performed in triplicate, as independent biological experiments. Plating efficiencies for the cells were as followed: - HCT116 (45%), HT29 (40%) and LIM1215 (30%). Statistical analysis was performed using the CFAssay for R package [24]. For the analysis of cell survival curves, the CFAssay uses the linear-quadratic model (LQ model) to compare responses to radiation in the presence of an intervention versus a respective control.

### 2.6. Necrosis Analysis

Cells were stained with 4 μg/mL propidium iodide and 1 μg/mL Hoechst 33,342 in triplicate for 10 min at room temperature before imaging using an EVOS confocal microscope (Life Technologies, Paisley, UK). All experiments were performed in triplicate. Apoptotic cells show an increased uptake of Hoescht 33,342 due to membrane permeability. Propidium iodide (PI) discriminates early apoptotic cells with dye exclusion due to intact cell membrane and necrotic cells which have loss of membrane integrity.

### 2.7. Flow Cytometry Analysis

Cells were trypsinized, pelleted at 100× *g* for 5 min at 4 °C using ice cold PBS along with the existing media to ensure no cell loss, and resuspended in 500 µL of Annexin V (1:20,000, diluted with Annexin binding buffer) and 0.5 µL of PI (0.5 µg/mL) for 20 min at room temperature whilst being protected from the light. Apoptosis analysis was performed by flow cytometry using the Attune NxT Flow Cytometer (Life Technologies, Paisley, UK).

### 2.8. CRISPR Gene Editing of asah1

Two guides targeting *asah1* (guide 321: 5′-GCTCAAGCTCACTCACCGG-3′ and guide 421: 5′-TAGCAGCCAACGCCACTCCC-3′) were designed using CRISPOR (Concordet and Haeussler, 2018) and purchased from Integrated DNA Technologies (IDT, Leuven, Belgium). The individual guides were cloned into pLenti-CRISPRv2 (Feng Zhang, Broad Institute; Addgene plasmid #52961) and the resulting plasmids were transfected into Lenti-X 293T cells together with psPAX2 and pMD2.G using standard PEI transfection techniques. After 72 h, the supernatant was collected and lentivirus particles were concentrated by ultracentrifugation at 100,000× *g* for 2 h. The purified lentiviral particles were combined and transduced into HCT116 cells, which were incubated overnight at 37 °C at 5% CO_2_. Following a change of media, cells were then incubated for 72 h and subjected to puromycin selection (1 µg/mL for 72 h) before undergoing the first genotyping screen using *asah1* screening primers (5′-GCCCAGCACGAGGTGTTCCT-3′, 5′-TCGGTCCGACTATTGCCCGC-3′). This genotyping indicated the presence of deletions corresponding to what would be expected using guides 321 and 421. The cells were subject to limiting dilution, and incubation continued for ~2 weeks. Resulting clones were subject to further genotyping to confirm the presence of the deletion.

### 2.9. Organoid Viability Assays in Response to Irradiation

Organoids were kindly gifted by the Wellcome Sanger Institute (Cambridge, UK) derived as part of the Cell Model Network UK and Human Cancer Models Initiative; HCM-SANG-0265-C18 (referred to as COLIMV005), HCM-SANG-0266-C18 (referred to as COLO005), HCM-SANG-0280-C18 (referred to as COLO081), COLO109 and COLO131. 10,000 cells from dissociated organoids were embedded in 30 μL of matrigel in a 96-well plate and allowed to mature. A single dose of radiation (2 Gy) was delivered via a CellRad X-ray irradiator (Faxitron Bioptics, Tucson, AZ, USA). Organoids were allowed to recover for 5–8 days and cell viability assessed using the CellTiter-Blue (Promega). Data were normalised to the unirradiated control to calculate viability of the irradiated cells [25]. All experiments were performed in triplicate, as independent biological experiments.

## 3. Results

### 3.1. Correlation of Acid Ceramidase Protein Expression and Colorectal Cancer Cell Radiosensitivity

**Figure 1:**
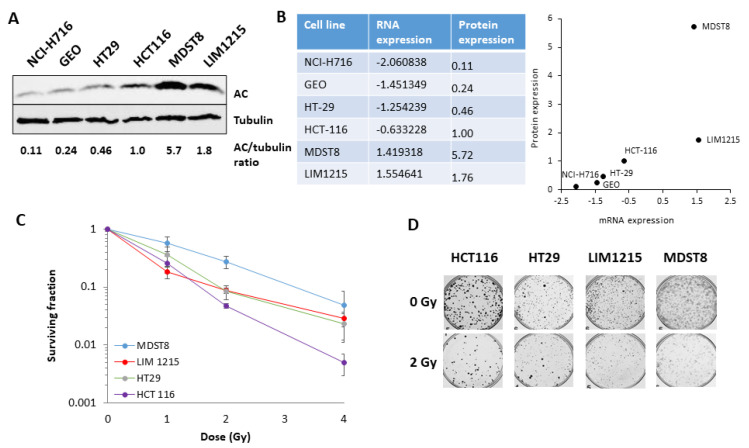
Protein levels of acid ceramidase (AC) in colorectal cancer cells show good correlation with the degree of radiosensitivity. (**A**) AC protein expression in whole cell extracts from colorectal cancer cells was analysed by immunoblotting, along with tubulin as a loading control. (**B**) mRNA and protein expression levels of AC in colorectal cancer cells, which is also shown graphically. (**C**) Radiosensitivity of colorectal cancer cells was analysed by clonogenic assays. (**D**) Representative images of colonies in non-irradiated irradiated (2 Gy) plates are shown (the latter were seeded with four times the number of cells).

Analysis of protein expression of AC in six colorectal cancer cell lines (NCI-H716, GEO, HT29, HCT116, MDST8 and LIM1215) showed variable levels (Figure 1A). Protein expression was generally in keeping with RNA expression (Figure 1B). We then examined comparative radiosensitivity of the cell lines using clonogenic assays, although two cell lines (NCI-H716, GEO) grown in suspension were omitted. MDST8 cells, with the highest AC protein expression, displayed the greatest cell survival post-irradiation, and therefore, radioresistance (Figure 1C,D). Interestingly, LIM1215 reproducibly demonstrated reduced survival at 1 Gy, but were generally radioresistant at higher radiation doses along with HT29 cells. HCT116 cells were the most radiosensitive. This was confirmed by statistical analysis (CFAssay [24]) that demonstrated significantly increased radiosensitivity of HCT116 compared to HT29, LIM1215 and MDST8 cell lines (Table 1).

### 3.2. Changes in AC Expression Modulate Radiosensitivity of Colorectal Cancer Cells

**Figure 2:**
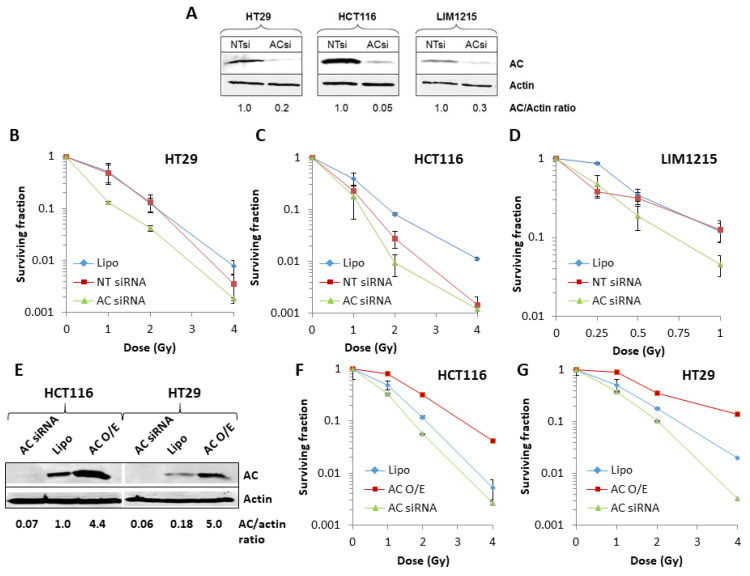
Modulation of AC expression controls the radiosensitivity of colorectal cancer cells. (**A**) AC protein expression in whole cell extracts from HT29, HCT116 and LIM1215 cells following AC or non-targeting (NT) siRNA was analysed by immunoblotting, along with actin as a loading control. (**B**–**D**) HT29, HCT116 or LIM1215 cells were treated with transfection reagent (lipofectamine) only, AC siRNA or a non-targeting control (NT) siRNA knockdown for 48 h, and radiosensitivity measured using clonogenic assays. (**E**) AC protein expression in whole cell extracts from HCT116 and HT29 cells following transfection reagent (Lipofectamine) only, AC siRNA for 48 h, or a mammalian expression plasmid for AC (AC O/E) for 24 h was analysed by immunoblotting, along with actin as a loading control. (**F**,**G**) HCT116 or HT29 cells were treated with transfection reagent (lipofectamine) only, AC siRNA for 48 h or a mammalian expression plasmid for AC (AC O/E) for 24 h and radiosensitivity measured using clonogenic assays.

To directly demonstrate the role of AC in modulating colorectal cancer cell radiosensitivity, we firstly depleted the enzyme using siRNA, which was shown to be >70% effective in HCT116, HT29 and LIM1215 cells (Figure 2A). On analysis of cell survival, reduced radiation doses were used for LIM1215 to account for prior variability in colony formation at higher doses. We observed statistically significant increases in radiosensitivity of HT29 (*p* < 0.00001), LIM1215 (*p* < 0.002) and HCT116 (*p* < 0.03) cells when AC was depleted using siRNA in comparison to non-targeting control siRNA treated cells (Figure 2B–D, Figure S1A–C and Table 2). siRNA knockdown of AC in MDST8 cells led to significant post-radiation cell death, even at low dosing (data not shown). Note that the non-targeting (NT) control siRNA also appeared to have an impact on cell survival post-irradiation, particularly in HCT116 cells, as compared to the transfection reagent (Lipofectamine) only. We then increased the protein levels of AC through a mammalian expression plasmid, where a 4.4–5.0-fold protein increase was observed in HCT116 and HT29 cells, respectively (Figure 2E). On analysis of cell survival post-irradiation, we found that HCT116 cells were significantly more radioresistant compared to both lipofectamine control (*p* < 0.004) and ASAH1 siRNA knockdown across irradiation doses (Figure 2F, Figure S1A–C and Table 2). Similarly, overexpression of AC was found to significantly (*p* < 0.00001) increase the radioresistance of HT29 and LIM1215 cells (Figure 2G, Figure S1A–C and Table 2). These data demonstrate that AC plays a significant role in controlling radiosensitivity of colorectal cancer cells.

### 3.3. CRISPR AC Knockout Confers Radiosensitivity for HCT116 Colorectal Cancer Cell Line

**Figure 3:**
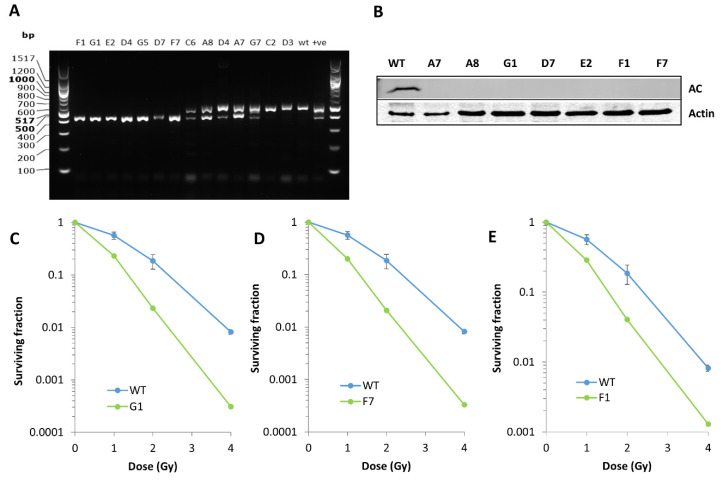
CRISPR gene editing of *asah1* leads to enhanced radiosensitivity of HCT116 colorectal cancer cells. (**A**) *Asah1* PCR genotyping of CRISPR clones confirms complete gene deletions in clones with a single DNA band at 400 bp (F1, G1, E2, D4, G5, D7, F7), single allele deletions for clones displaying DNA bands at 400 and 500 bp (C6, A8, D4, A7 and G7) and unsuccessful deletion for clones with a DNA band at 500 bp only (C2, D3), in keeping with a WT-expressing *asah1* clone. (**B**) AC protein expression in whole cell extracts from CRISPR clones compared to a WT control was analysed by immunoblotting using AC antibodies, along with actin as a loading control. (**C**–**E**) *Asah1* CRISPR knockout clones (G1, F7 and F1) were treated with escalating doses of radiation (1–4 Gy) and cellular radiosensitivity compared to the WT *asah1*-expressing control, assessed using clonogenic assays.

To further demonstrate the role of AC in modulating colorectal cancer cell radiosensitivity, we targeted *asah1* using CRISPR gene editing within the HCT116 cell line. The genotyping for individual clones was analysed using PCR to assess single or double allele deletion. Clones labelled F1, G1, E2, D4, G5, D7 and F7 yielded a single band of approximately 400 bp following genotyping PCR, in keeping with a complete *asah1* deletion (Figure 3A). Clones C6, A8, D4, A7 and G7 displayed two bands, the second at 500 bp in keeping with a wild type (WT) control, and therefore indicated a single allele deletion only. Clones C2 and D3 had only a single band at 500 bp indicating no successful deletion (Figure 3A). Immunoblotting was performed on cell extracts derived from the A7, A8, G1, D7, E2, F1 and F7 clones, which confirmed a lack of AC protein expression for both the double (G1, D7, E2, F1 and F7) and surprisingly the single (A7 and A8) allele knockouts (Figure 3B). Focusing specifically on the complete gene knockouts, and on analysis of cell survival post-irradiation, we found that *asah1* CRISPR cell lines were significantly more radiosensitive compared to asah1-expressing WT control cells (Figure 3C–E) across clones; G1 (*p* < 0.000001), F7 (*p* < 0.000001), F1 (*p* < 0.000001). These findings were consistent across each successful CRISPR clone (data not shown). These data further demonstrate that AC protein expression plays a significant role in controlling radiosensitivity of colorectal cancer cells.

### 3.4. Correlation of AC Protein Expression and Rectal Cancer Organoid Radiosensitivity

**Figure 4:**
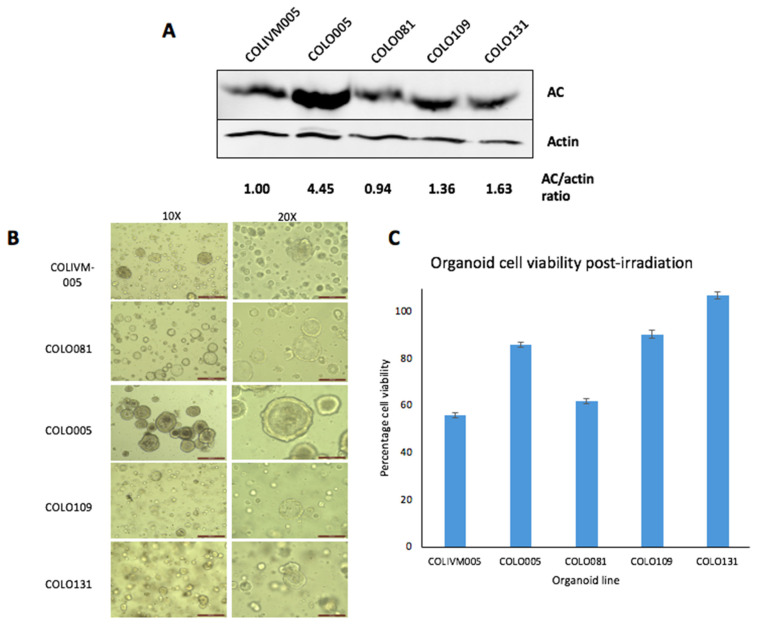
Increased expression of AC expression in colorectal organoids is associated with increased resistance to radiation. (**A**) Western blotting demonstrating varying levels of baseline AC expression (**B**) Representative images of primary organoids at 10× and 20× magnification during growth (**C**) Organoids were treated with a single dose of radiation (2 Gy) and viability assessed after 5–8 days.

Colorectal cancer primary tissue organoids were sourced (Dr. Hayley Francies, Wellcome Sanger Institute, Cambridge, UK) containing either low or high *asah1* mRNA expression and which was confirmed by immunoblotting, as shown by a 1.36–4.45 AC/actin ratio in high expressors versus those containing low AC (0.94–1.00 AC/actin ratio) (Figure 4A). Organoids were embedded in matrigel and allowed to grow for 7–11 days between passages to develop their characteristic appearances (Figure 4B). Organoids were treated with a single radiation dose (2 Gy) in a 96-well plate and cell viability was analysed after 5–8 days compared to unirradiated control organoids. We observed that organoids with a relatively high baseline AC expression (COLO005, COLO109, COLO131) had significantly increased viability, and therefore were more radioresistant, compared to those with a lower AC expression (COLIVM005, COLO081) (Figure 4C and Table 3).

### 3.5. Pharmacological Inhibition of AC Improves Radiosensitivity of Colorectal Cancer Cells

**Figure 5:**
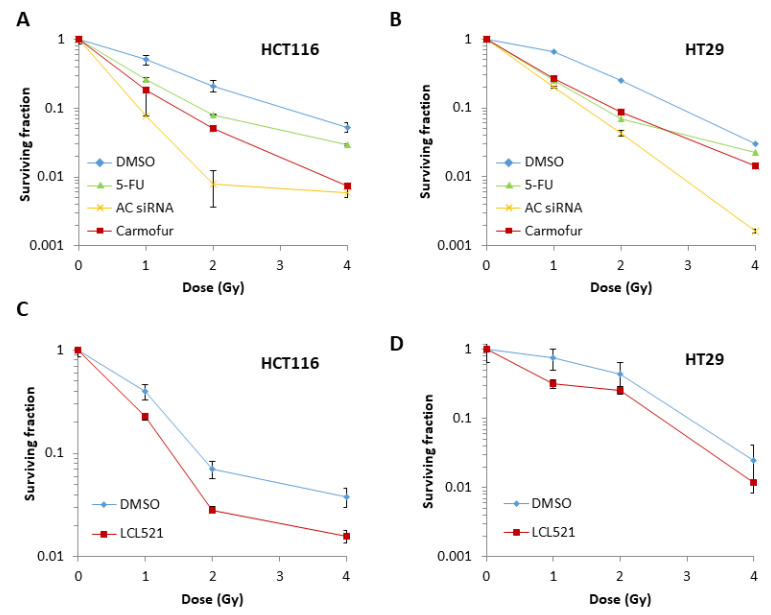
Pharmacological inhibition of AC expression increases the radiosensitivity of colorectal cancer cells. (**A**) HCT116 or (**B**) HT29 cells were pretreated with a vehicle control (DMSO), 5 µM 5-fluorouracil (5-FU), or 4 µM carmofur for 2 h, or with AC siRNA for 48 h, and radiosensitivity measured using clonogenic assays. (**C**) HCT116 or (**D**) HT29 cells were pretreated with a vehicle control (DMSO), or 10 µM LCL521 for 2 h, and also for a further 24 h post-plating, and radiosensitivity was measured using clonogenic assays.

We compared the radiosensitivity of colorectal cancer cells in combination with 5-FU, a non-specific pharmacological inhibitor of AC (carmofur), in addition to a specific AC inhibitor (LCL521). Using an ELISA assay, we found that AC detection was reduced in HT29 cells by >80% with 4 μM carmofur, whilst 8 μM carmofur was required for the same level in HCT116 cells (Figure S2A,B). To limit off-target effects, particularly in HCT116 cells, the dose was reduced to 4 μM carmofur (~55% reduction in AC detection). HCT116 cells showed increased radiosensitivity in the presence of carmofur and 5-FU (Table 4 and Figure S5A), which were less effective in comparison to siRNA knockdown of AC (Figure 5A). Similarly, HT29 cells were significantly radiosensitised in the presence of carmofur and 5-FU (Table 4 and Figure S5A), whereas the impact of AC siRNA appeared to be relatively more effective in reducing cell survival (Figure 5B). On utilizing the specific inhibitor for AC, LCL521, an ELISA assay demonstrated a >80% reduction in AC detection in both HCT116 and HT29 cells using 10 μM LCL521 (Figure S5A,B). We demonstrate that LCL521 significantly increased the radiosensitivity of HT29 cells (*p* < 0.03), and also enhanced the radiosensitivity of HCT116 cells although this was not statistically significant compared to a vehicle only (DMSO) control (Figure 5C,D, Figure S3A,B and Table 4).

### 3.6. Inhibition of AC Increases Apoptosis Post-Irradiation in a p53-Dependent Manner

**Figure 6:**
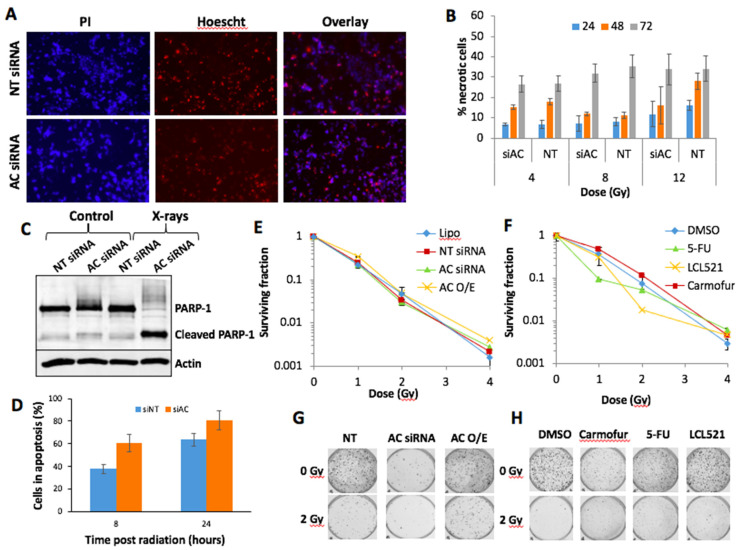
Reduction in survival of colorectal cancer cells through inhibition of AC is caused by apoptosis. (**A**,**B**) HT29 cells were treated with AC siRNA or a non-targeted control (NT) for 48 h prior to irradiation and stained at 24 h intervals with Hoescht 33,342 and propidium iodide stain. (**A**) Representative images of cells irradiated with 12 Gy and (**B**) percentage of necrotic cells at 24–72 h post-irradiation with 4–12 Gy. (**C**) PARP-1 protein expression, and PARP-1 cleavage, in whole cell extracts from HT29 cells in the absence and presence of 4 Gy radiation after 24 h was analysed by immunoblotting, along with actin as a loading control. (**D**) HCT116 cells treated with AC siRNA and a non-targeted control siRNA after 8 or 24 h post irradiation (4 Gy) were analysed by flow cytometry to determine levels of cellular apoptosis (**E**) p53^−/−^ HCT116 cells were treated with transfection reagent (lipofectamine) only, AC siRNA or a non-targeting control (NT) siRNA knockdown for 48 h, or a mammalian expression plasmid for AC (AC O/E) for 24 h and radiosensitivity measured using clonogenic assays. (**F**) p53^−/−^ HCT116 cells were treated with a vehicle control (DMSO), 5 µM 5-FU, or 4 µM carmofur for 2 h, or with LCL521 for 24 h, and radiosensitivity measured using clonogenic assays. (**G**,**H**) Representative images of colonies in non-irradiated irradiated (2 Gy) plates are shown (the latter were seeded with 2 times the number of cells).

To examine the mechanism of increased radiosensitivity of colorectal cancer cells in the absence of AC, we analysed the levels of apoptotic and necrotic cells. Using a 4–12 Gy dose of radiation to stimulate cell death, we demonstrate that there are no significant increases in the levels of necrotic HT29 cells at 24–72 h post-irradiation following AC siRNA knockdown in comparison to a non-targeting control siRNA (Figure 6A,B). On immunoblotting analysis of PARP-1 cleavage, as a marker of apoptosis, siRNA knockdown independent of AC in HT29 cells appeared to cause a minimal increase in PARP-1 cleavage, compared to a non-targeting control siRNA (Figure 4C; compare lanes one and two). However, in response to radiation, there was dramatic PARP-1 cleavage (and associated PARP-1 poly(ADP-ribosylation)) in AC siRNA-treated cells, which was not observed in non-targeting control siRNA-treated cells (Figure 6C; compare lanes three and four). This demonstrates that the mechanism of cell death in these cells is through apoptosis. This is supported by flow cytometry analysis which confirmed a significantly higher level of apoptosis in AC siRNA-treated cells compared with a non-targeting control siRNA at both 8 (*p* < 0.01) and 24 h (*p* < 0.02) post-irradiation (Figure 6D). It is well established that the tumour suppressor protein p53 is essential for cells to undergo apoptosis. We therefore analysed the impact of AC expression on the radiosensitivity of a p53^−/−^ HCT116 cell line. We observed that an siRNA knockdown of AC, or overexpression of AC, had no impact on the radiosensitivity of these p53-deficient cells using clonogenic assays (Figure 6D,F and Figure S5). We also found no statistically significant difference in the radiosensitivity of these cells to 5-FU, carmofur or LCL521 (Figure 6E,G and Figure S5). This demonstrates that the decrease in cell survival post-irradiation following inhibition of AC is achieved via apoptosis in a p53-dependent manner.

## 4. Discussion

Colorectal cancer is the second commonest cause of cancer related death in the UK, and rectal cancer accounts for approximately 25% of these cases. Surgical resection of advanced rectal cancer represents a particular surgical challenge due to the anatomical confines of the bony pelvis and adjacent neurovascular structures. Neoadjuvant CRT is employed widely in order to reduce the tumour bulk and improve the chances of obtaining a clear surgical resection margin, however, the effects are still very variable and unpredictable. Those patients who do experience a complete clinical response have a more favorable long-term prognosis, and may also be candidates for organ preserving treatment, although the correlation between complete clinical (radiological) and pathological (post resection) response is unclear. CRT is not without complications, both surgically and also in terms of long-term anorectal function in those patients undergoing resection. Furthermore, the delay to systemic treatment introduced with CRT means that that those patients who respond poorly are at increased risk of long-term treatment failure. Biomarker studies in rectal cancer are thus desperately needed both to identify those patients that will undergo good responses and those that will not, so that unnecessary delays to systemic treatment can be avoided. Given the favorable prognosis associated with complete pathological response (~12%), therapeutic biomarker studies that might increase this rate are also highly desirable.

**Figure 7:**
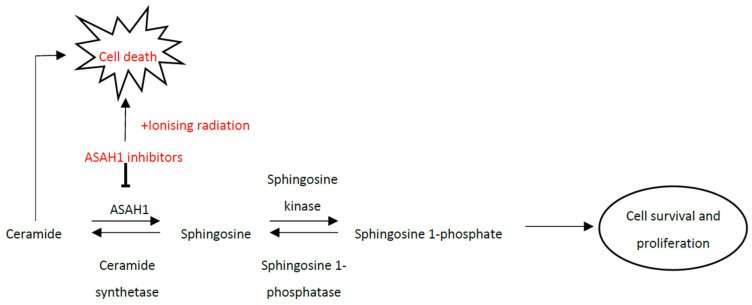
A schematic diagram summarizing the ceramide metabolism pathway and effects of acid ceramidase (ASAH1) manipulation.

We have recently identified a novel finding that AC is associated with a poor response to CRT in patients with advanced rectal cancer. Whilst the initial exploratory work analysed the pre-treatment biopsies of patients, the validation work analysed only surgical resection specimens and thus its utility as a predictive biomarker is unknown. Overexpression of AC in human cancers is well described in a variety of cancers, of particular relevance to this study are prostate, CNS and head and neck cancers, where manipulation of AC has been shown similarly to influence chemo and radiosensitivity through pro-apoptotic pathways [10,11,12,13,14] (Figure 7). However, there has been little work exploring the role of AC in rectal cancer. In this work, we have now explored whether acid AC may represent a therapeutic biomarker to increase the radiosensitivity of rectal cancer. We have employed standard colorectal cancer cell lines which are routinely used for investigation of rectal cancer response to radiotherapy, however we advanced our work by also utilising patient-derived rectal cancer 3D organoid models.

We have confirmed that there is differential expression of AC across our 2D colorectal cancer cell lines (HCT116, HT29 and LIM1215) and 3D organoid models, but that higher expression largely correlates with increased radioresistance in these ex vivo models. We have shown that biological (both siRNA and CRISPR) and pharmacological (carmofur and LCL521) inhibition of AC in 2D cell lines increases radiosensitivity, which appears to be consistent across a number of different colorectal cancer cell lines. Conversely, overexpression of AC enhanced cellular radioresistance, confirming the direct role for AC in modulating radiosensitivity. We discovered that enhanced radiosensitivity of AC knockdown cells was caused by increases in apoptosis, as demonstrated by PARP-1 cleavage and flow cytometry analysis, and that this mediated in a p53-dependent manner due to the lack of change in radiosensitivity of p53-deficient HCT116 cells through AC modulation. Noteworthy, is that HCT116 and LIM1215 cells both harbor wild type p53, whereas HT29 cells contain mutant p53 but which is still radiosensitised by AC knockdown/inhibition. Nevertheless, this correlates with recent work demonstrating that ceramide, the cellular target for degradation by AC, binds to p53 leading to its cellular accumulation and stress response activation through disruption of interaction with the E3 ubiquitin ligase MDM2 that targets p53 for degradation [25]. Ceramides have long been known to be mediators of apoptosis in response to stimuli, including tumour necrosis factor α and Fas ligand, and which is triggered through effector protein kinases including JNK and the pro-apoptotic Bcl-2 family members Bid and Bad [26,27]. Therefore, our work strengthens the importance of AC and the ceramide pathway in controlling cell survival in response to exogenously-induced stress, but more importantly that AC is a cellular target for radiosensitisation in colorectal cancer cells.

In this study we employed two pharmacological AC inhibitors. Carmofur (1-hexylcarbamoyl-5-fluoruracil) has been in clinical use in the adjuvant setting since 1981, where it appears to augment the disease free and overall survival in breast, gastric, bladder and colon cancers [28]. Carmofur is a 5-FU oral prodrug, however until recently, the mechanism of its independent 5-FU anti-tumour activity were unknown. Recent work has, however, confirmed that Carmofur does directly bind to AC [29], and this may explain it synergistic effects with chemotherapy agents, such as 5-FU and oxaliplatin [30]. Unfortunately, Carmofur may be limited use in a wider clinical context due to its potential CNS side effects of leukoencephalopathy, which may explain its restricted use [16].

In view of this, we chose to explore the effects of a specific small molecular inhibitor of AC, LCL521. LCL521 is a prodrug of the AC inhibitor B13, which has been designed to provide efficient lysosomal delivery of the drug, which shows more effectiveness in inhibiting AC compared to non-compartmentalized drugs [18]. Our results suggested that LCL521 was associated with an increase in radiosensitivity in the HT29 and HCT116 cell lines. However, this effect was not statistically significant in HCT116 cells, which may be due to the fact that we were only able to achieve an ~80% reduction in AC detection at a 10 µM dose of LCL521, in line with other studies [31]. Although LCL521 has not yet been evaluated in the clinical setting, there is an increasing interest in developing drugs that act on the sphingolipid pathway. The biological data demonstrate the impact of AC manipulation on radiosensitivity as a proof of principle, and therefore, its potential as a clinical target. However, further research into the identification of more potent and effective drug(s) or an siRNA therapy would be required to address inter-tumour variability in response to radiation, in comparison to that demonstrated with our pharmacological data using LCL521. Proposed mechanisms through which this will be achieved include nanotechnology drug delivery, along with more traditional targeted antibodies [32,33]. These mechanisms are similar to the ones we have demonstrated with pharmacological inhibition, which ultimately will be more transferrable to clinical practice.

## 5. Conclusions

In conclusion, this work continues to highlight the importance of the sphingolipid pathway in cancer, in particular, the role that AC may play in mediating the response of rectal cancer to radiotherapy. Further work with more advanced pre-clinical models (patient-derived organoids, but more so mouse models) are required to confirm our in vitro findings, along with novel drug development and/or delivery that will allow the accurate compartmental delivery of agents, such as LCL521.

## Figures and Tables

**Table 1:** Comparative survival of colorectal cancer cells in response to X-ray radiation.

Cell Line	Comparative Cell Line	Statistical Analysis
HCT116	HT29	*p* < 0.0003
HCT116	LIM1215	*p* < 0.000002
HCT116	MDST8	*p* < 0.000001

**Table 2:** Comparative survival of colorectal cancer cells with modified levels of AC in response to X-ray radiation.

Comparative Treatment	HCT116	HT29	LIM1215
NT siRNA vs. AC siRNA	*p* < 0.03	*p* < 0.00001	*p* < 0.002
Lipo vs. AC siRNA	*p* < 0.00001	*p* < 0.0001	*p* < 0.0004
Lipo vs. AC O/E	*p* < 0.004 *	*p* < 0.00001 *	*p* < 0.00001 *

* Denotes increased radioresistance.

**Table 3:** Comparative cell viability of colorectal cancer organoids in response to 2Gy X-ray radiation.

Organoid Line	Comparative Organoid Line	Statistical Analysis
COLIVM005	COLO005	*p* < 0.03
COLIVM005	COLO109	*p* < 0.05
COLIVM005	COLO131	*p* < 0.001
COLO081	COLO005	*p* < 0.001
COLO081	COLO109	*p* < 0.02
COLO081	COLO131	*p* < 0.002

**Table 4:** Comparative survival of colorectal cancer cells following drug treatments in response to X-ray radiation.

Comparative Treatment	HCT116	HT29
DMSO vs. carmofur	*p* < 0.00006	*p* < 0.05
DMSO vs. 5-FU	*p* < 0.00001	*p* < 0.00001
DMSO vs. LCL521	*p* = 0.18	*p* < 0.03

## Supplementary Materials

The following are available online at https://www.mdpi.com/2073-4409/9/12/2693/s1, Figure S1: Biological manipulation of AC effects colony formation efficiency post-irradiation; Figure S2: ELISA assay demonstrating effect of Carmofur dosing on AC detection; Figure S3: Pharmacological targeting of AC effects colony formation efficiency post-irradiation; Figure S4: ELISA assay demonstrating effect of LCL521 dosing on AC detection; Figure S5: Biological and pharmacological targeting of AC in HCT116 p53^−/−^ cells does not affect colony formation efficiency post-irradiation.
