# Supplementary material for: Targeting Acid Ceramidase to Improve the Radiosensitivity of Rectal Cancer
Source: Cells. 2020 Dec 15;9(12):2693. doi: 10.3390/cells9122693 (PMC7765421; doi:10.3390/cells9122693)
Supplement: Supplementary file 1 [file cells-09-02693-s001.pdf]

cells MDPI

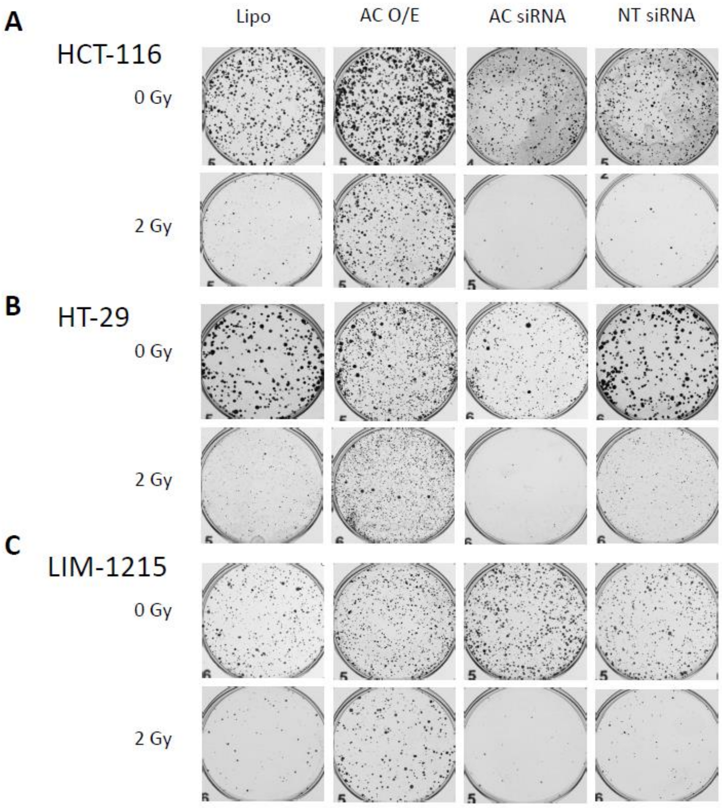


**Figure S1.** Biological manipulation of AC effects colony formation efficiency post-irradiation.

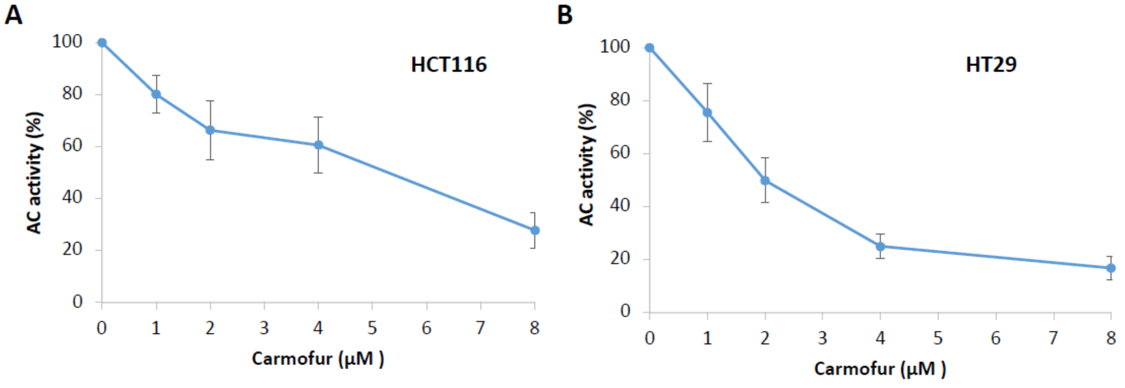


**Figure S2.** ELISA assay demonstrating effect of Carmofur dosing on AC detection.

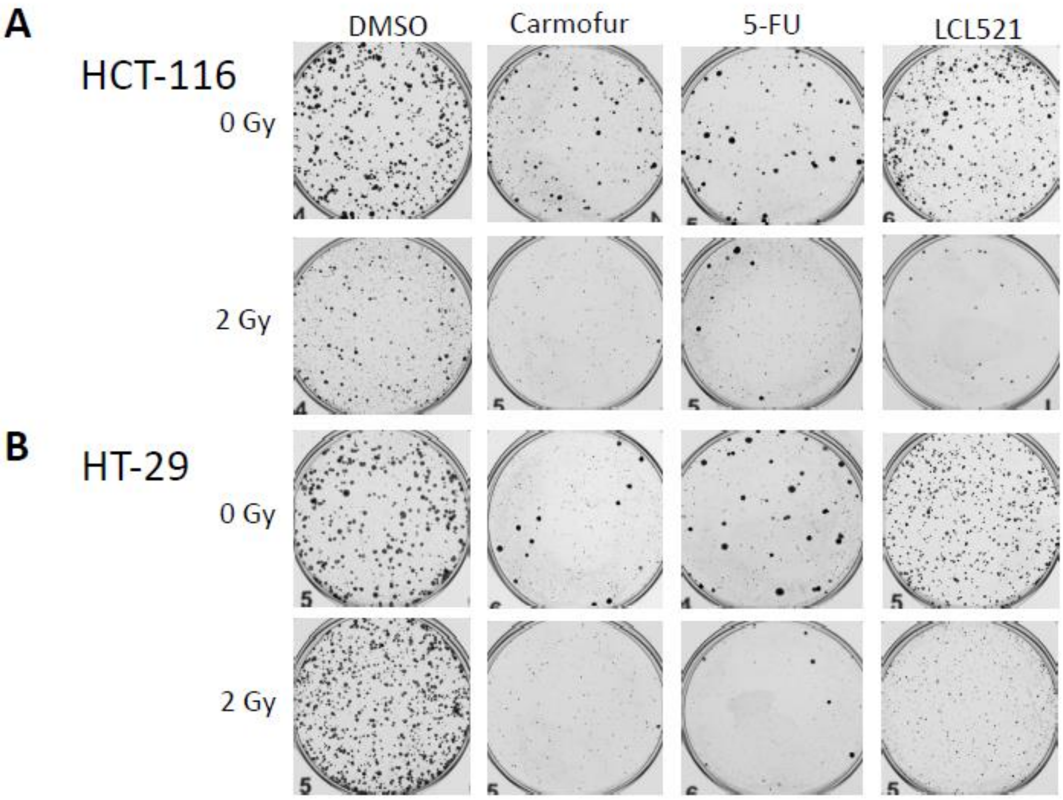


**Figure S3.** Pharmacological targeting of AC effects colony formation efficiency post-irradiation.

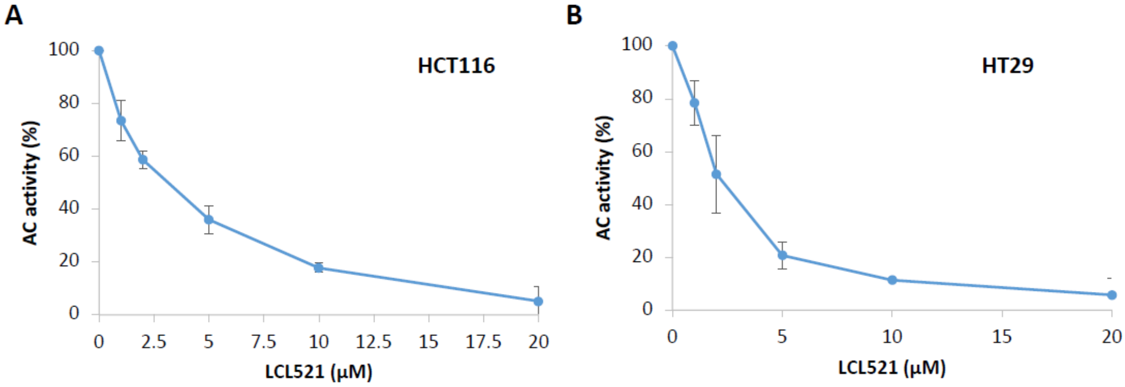


**Figure S4.** ELISA assay demonstrating effect of LCL521 dosing on AC detection.

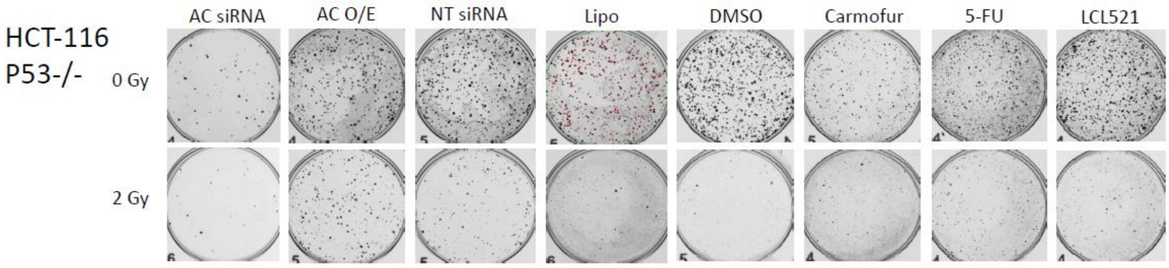


**Figure S5.** Biological and pharmacological targeting of AC in HCT116 p53$^{-/-}$ cells does not effect colony formation efficiency post-irradiation.
